# Effects of* Cymbidium* Root Ethanol Extract on Atopic Dermatitis

**DOI:** 10.1155/2016/5362475

**Published:** 2016-02-11

**Authors:** Wan-Joong Kim, Hae-Sim Cha, Myung-Hun Lee, Sun-Young Kim, Seo Ho Kim, Tack-Joong Kim

**Affiliations:** Division of Biological Science and Technology, Yonsei-Fraunhofer Medical Device Laboratory, College of Science and Technology, Yonsei University, Wonju 220-710, Republic of Korea

## Abstract

*Cymbidium* has known antibacterial and antiedema activity and has been used as an ingredient in cosmetics and fragrances. The effects of* Cymbidium* ethanol extract (CYM) on allergic response and the underlying mechanisms of action have not been reported. Therefore, the purpose of this study was to determine the effect of CYM on allergic responses. Topical application of CYM was effective against immunoglobulin E (IgE)/dinitrophenyl-conjugated bovine serum albumin- (DNP-BSA-) induced degranulation of RBL-2H3 cells and anaphylaxis in ICR mice. An allergic dermatitis-like mouse model was used to evaluate the therapeutic potential of CYM* in vivo. *Continuous application of 2,4-dinitrochlorobenzene (DNCB) not only induced dermatitis in ICR mice but also aggravated the skin lesioning. However, the application of CYM decreased skin lesion severity, scratching behavior, and IgE levels. In addition, CYM downregulated the expression of the proinflammatory cytokines interleukin- (IL-) 4, IL-13, and tumor necrosis factor- (TNF-) *α*. Studies of signal transduction pathways showed that CYM suppressed the phosphorylation of spleen tyrosine kinase (Syk), an upstream molecule. It also inhibited the phosphorylation of Akt, phospholipase C- (PLC-) *γ*, and mitogen-activated protein kinase kinase kinase (MEKK). These results indicate that CYM may be effective in preventing and reducing allergic response and may have therapeutic potential as an antiallergic agent in disorders such as atopic dermatitis.

## 1. Introduction

An allergic response is a hypersensitive immune reaction to a diverse and complex combination of environmental and genetic factors [1–3]. It occurs in various diseases such as atopic dermatitis (AD), anaphylactic shock, asthma, and conjunctivitis [4, 5]. AD is a chronic inflammatory skin disease affecting approximately 10 million people, who are also prone to experiencing relapses. AD is accompanied by pruritus, dry skin, eczema, and keratinization [6]. It is also induced by complicated conditions and mechanisms that are closely related to increased T helper 2 lymphocytes and basophils [7, 8] while activated mast cells, increased immunoglobulin E (IgE), and production of proinflammatory cytokines are found in the skin lesions [8, 9]. Mast cells play a key role in the allergic response in conditions such as AD, food allergy, allergic rhinitis, and other similar diseases by activating a tetrameric form of the Fc epsilon receptor 1 (Fc*ε*R1) [9, 10]. Fc*ε*R1 is also expressed in basophils and antigen-presenting dendritic cells and consists of *α*, *β*, and *γ* chains. The *α* chains directly bind the IgE heavy chains while *β* and *γ* chains are involved in signal transduction [11, 12]. The IgE produced and secreted by plasma B cells has a high affinity for IgE-specific Fc*ε*R1 on mast cells [13–15]. When exposed to a second IgE-specific allergen, signaling pathways in mast cells are initiated by IgE allergen cross-linking. Tyrosine kinases are phosphorylated and activated by cross-linking of Fc*ε*R1 by *β*- and *γ*-receptor tyrosine-based activation motifs [16]. Phosphorylation of Lyn and spleen tyrosine kinase (Syk) activates various downstream signaling molecules such as protein kinase B (Akt), phospholipase C- (PLC-) *γ*, and mitogen-activated protein kinase kinase kinase (MEKK) as well as PLC-*γ*-mediated cleavage of phospholipid phosphatidylinositol 4,5-bisphosphate (PIP2) to diacylglycerol (DAG) and inositol 1,4,5-trisphosphate (IP3) [1, 17]. IP3 increases calcium ion influx into the cytosol through calcium channels [18, 19]. Degranulation of mast cells is induced by increased calcium ions in the cytosol and activation of PKC.

Following mast cell degranulation, various factors are released such as histamine, leukotrienes, prostaglandins, and other mediators that induce smooth muscle contraction, deposition of collagen and other tissues, and vasodilation [20, 21]. In addition, the activation of MEKK promotes various downstream signal transduction pathways. Mitogen-activated protein kinases (MAPKs) are downstream and regulate the production of cytokines such as interleukin- (IL-) 4, IL-13, and tumor necrosis factor- (TNF-) *α*. These cytokines exert proinflammatory actions associated with the allergic response [22, 23].

*Cymbidium* belongs to the orchid family, which is distributed throughout Southeast Asia, China, Japan, and Northern Australia. Various research studies have demonstrated that phenanthrene and phenylpropanoid extracted from the roots of* Cymbidium* exert antibacterial effects against hay bacillus, pneumobacillus, and* Trichophyton rubrum* [24].* Cymbidium* has shown anti-inflammatory effects in a mouse model of carrageenan-induced edema [25]. Linalool and 4-methyl-phenol, which are isolated from the roots of* Cymbidium* by chromatography, are used as ingredients in cosmetics and fragrances [26]. Aromatic glucosides from* Cymbidium* extracts also have antioxidant potential [27]. The antiallergy effects of* Cymbidium* root extracts and their mechanisms of action have not been reported. Therefore, we investigated the effects of the* Cymbidium* ethanol extract (CYM) on allergic responses* in vitro* and* in vivo.*

## 2. Material and Methods

### 2.1. Preparation of CYM

*C. eburneo-kanran* was cultivated according to the agricultural practices and methods of the Korea Rural Development Administration and harvested in Eumseong (GPS: E 128°62′ N 36°56′). For extract preparation, 400 g of dried* C. eburneo-kanran* root was extracted thrice with 2 L of ethanol for 1 day. The resulting extracts were filtered through Whatman No. 1 paper, combined, and concentrated using a rotary evaporator (EYELA N-1000, Tokyo, Japan) at 40°C. The acquired dried extracts were subsequently lyophilized and processed into powder.

### 2.2. Cell Culture

RBL-2H3 is a rat cell line and was acquired from the American Type Culture Collection (ATCC, Rockville, MD, USA). The cells were cultured in Eagle's Minimal Essential Medium (MEM, WelGENE, Inc., Daegu, Korea) supplemented with 100 units/mL penicillin-streptomycin (Sigma-Aldrich, St. Louis, MO, USA) and 10% (v/v) fetal bovine serum (FBS, Gibco, Rockville, MD, USA) at 37°C in a humidified 5% CO_2_ atmosphere. Trypsin-ethylenediaminetetraacetic acid (EDTA) solution (Sigma-Aldrich) was used to detach the cells, which were washed with phosphate-buffered saline (PBS, Gibco).

### 2.3. Animals

Seven-week-old male ICR mice were obtained from Orient Bio (Gangneung, Korea) and housed in cages maintained in an environment with the temperature at 20−22°C and humidity of 40−50%. All the ICR mice were given access to standard laboratory chow and water* ad libitum*. The Institutional Animal Care and Use Committee (IACUC, YWC-141128-1) at Yonsei University (Wonju, Korea) approved the protocol for this study.

### 2.4. *β*-Hexosaminidase Release Assay

RBL-2H3 cells (1 × 10^5^ cells/mL) were seeded into 24-well culture plates and incubated overnight in MEM containing 10% (v/v) FBS at 37°C. To sensitize RBL-2H3 cells, they were incubated with antidinitrophenyl (anti-DNP) IgE at 400 ng/mL. After 12 h, the sensitized RBL-2H3 cells were washed with Siraganian buffer (pH 7.2), containing 25 mM piperazine-N,N′-bis(2-ethanesulfonic acid) (PIPES), 4 mM potassium chloride (KCl), 0.4 mM magnesium chloride (MgCl), 110 mM sodium chloride (NaCl), 0.1% BSA, 5.6 mM glucose, 1 mM calcium chloride (CaCl_2_), and 40 mM hydrochloric acid (HCl), and were then treated with CYM (0, 25, 50, 100, or 200 *μ*g/mL) in Siraganian buffer at 37°C for 30 min. To activate RBL-2H3 cells, dinitrophenyl-conjugated- (DNP-) BSA, 50 ng/mL was added at 37°C for 15 min. Cell supernatants (30 *μ*L) were transferred to a 96-well culture plate and mixed with 1 mM p-nitrophenyl-N-acetyl-*β*-D-glucosaminide (p-NAG, MP Biomedicals, OH, USA) in 0.1 M citrate buffer for 1 h. To terminate the reaction, 0.1 M carbonate buffer was added, and the absorbance of the reaction solution was measured at 405 nm using a microplate reader.

### 2.5. Western Blotting

RBL-2H3 cells (1 × 10^5^ cells/mL) were seeded into 24-well plates and incubated in MEM containing 10% (v/v) FBS at 37°C for 12 h. Anti-DNP IgE (400 ng/mL) was added to the medium and incubated for 12 h. Then, the medium was replaced with serum-free MEM containing CYM (0 or 200 *μ*g/mL), followed by the addition of DNP-BSA. RBL-2H3 cells were washed twice with cold PBS and lysed, and protein was extracted using the PRO-PREP protein extraction kit (iNtRON Biotechnology, Inc., Seongnam, Korea) with a protease inhibitor containing 1 mM sodium vanadate (Na_3_VO_4_) and 10 mM sodium fluoride (NaF). A Bradford assay kit (BioRad, CA, USA) was used for the quantitative analysis of protein according to the manufacturer's instructions. The protein lysates were separated using 12.5% by sodium dodecyl sulfate-polyacrylamide gel electrophoresis (SDS-PAGE) and transferred to a polyvinylidene fluoride membrane (PVDF, Bio-Rad) using a Trans-Blot SD Semi-Dry Transfer Cell (Bio-Rad) for 40 min.

The PVDF membrane was blocked for 1 h with 5% skim milk in Tris-buffered saline containing 0.1% Tween-20 (TBS-T). The blocked membrane was incubated with primary antibody overnight at 4°C (1 : 2000). The primary antibodies used were anti-phospho-Syk, anti-Syk, anti-phospho-PLC-*γ*, anti-PLC-*γ*, anti-phospho-Akt, anti-Akt, anti-phospho-extracellular signal-regulated kinase (Erk) 1/2, anti-Erk 1/2, anti-phospho-c-Jun N-terminal kinase (JNK), anti-JNK, anti-phospho-p38, and anti-p38, which were purchased from Cell Signaling Technology (Danvers, MA, USA). After primary antibody incubation, the membrane was washed twice with TBS-T and incubated with the anti-rabbit IgG horseradish peroxidase- (HRP-) linked secondary antibody (1 : 5000, Cell Signaling Technology) for 2 h at room temperature. The signal detection was performed using the EZ-Western kit (DoGEN, Seoul, Korea), and the images were acquired and analyzed using an ImageQuant LAS4000 (GE Healthcare, Buckinghamshire, UK).

### 2.6. Passive Cutaneous Anaphylaxis

The anti-DNP IgE antibody was injected intradermally into each right ear of 7-week-old ICR mice. After 1 day, CYM or diphenhydramine (DPH) was orally administered to the mice and, 1 h later, 200 *μ*g of DNP-BSA in 200 *μ*L PBS containing 3% Evans Blue was injected into the tail vein. After 30 min, the mice were euthanized and their ears were excised and then incubated overnight with 500 *μ*L of formamide at 60°C. The amount of dye taken up was determined by measuring the absorbance at 620 nm using a microplate reader.

### 2.7. AD-Like Mouse Model

These experiments were performed according to the method of Park et al. [28] with slight modifications. Briefly, male ICR mice were divided into three groups of six animals each, the nontreatment, 2,4-dinitrochlorobenzene (DNCB) treatment, and DNCB and CYM treatment groups. AD was induced by shaving the dorsal skin of the mice and applying a 1% DNCB solution (3 : 1 mixture of acetone and olive oil) for 4 days. After sensitization, mice were treated with 200 *μ*L of 0.5% DNCB solution, and then the lesions on the dorsal skin were topically treated with 200 *μ*L of CYM (10 mg/mL) every 2 days for a total of six times. Subsequently, the AD-like mice were euthanized. We also evaluated the progression of the severity of the dorsal skin dermatitis of the AD-like ICR mice during treatment with 0.5% DNCB or 0.5% DNCB and CYM. Five conditions were evaluated, erythema, pruritus/dry skin, erosion, lichenification, and edema/excoriation. These conditions were then scored as follows based on severity: none = 0, mild = 1, moderate = 2, and severe = 3, and the sum of the individual scores was considered the dermatitis score. To ensure the credibility of the severity test, a researcher who was not associated with the experiment and was blinded to the treatments, determined the score. In addition, to observe scratching behavior, a behavioral characteristic of AD-like mice, we video-recorded and analyzed their behavior for 60 min during treatment with 0.5% DNCB or 0.5% DNCB and CYM. The number of scratches was counted in the video recordings by a similarly blinded researcher who was not associated with the study. Lastly, we histopathologically analyzed the skin by enucleating the dorsal lesions from mice in all three groups (untreated, DNCB treated, and DNCB and CYM treated); these lesions were fixed in 4% paraformaldehyde solution and cryoprotected in 30% sucrose solution. The skin samples were fixed in a cryomold using Tissue-Tek optimum cutting temperature (OCT) compound (Sakura Finetek Inc., CA, USA). The cryoprotected tissue samples were sectioned and stained with hematoxylin and eosin (H&E), and their thickness was measured using histopathological analysis with light microscopy (Nikon Eclipse TE2000-U, ACT-1 for DXM 1200, Japan).

### 2.8. Measurement of Serum IgE Levels

The serum IgE levels were measured using mouse blood collected from the heart. The serum was analyzed using a Mouse IgE enzyme-linked immunosorbent assay (ELISA) MAX*™* Deluxe (BioLegend, CA, USA) according to the manufacturer's instructions by measuring the standard and sample absorbance values at 450 nm using a microplate reader.

### 2.9. RNA Preparation and Real-Time Polymerase Chain Reaction (qPCR)

Total RNA was isolated from tissue extracted from the dorsal lesions using Tri-reagent (Sigma-Aldrich, St. Louis, MO, USA) according to the manufacturer's instructions. The concentration of total RNA was determined using a spectrophotometer (GE Healthcare). Total RNA was used as a template for cDNA synthesis, which was performed using a cDNA Synthesis kit (Takara Bio, Shiga, Japan). The real-time polymerase chain reaction (qPCR) analysis was carried out using SYBR Green I and a Lightcycler^®^ 96 instrument (Roche, Basel, Switzerland). The following primers were used: mouse IL-4, sense 5′-GGTCTCAACCCCCAGCTAGT-3′ and antisense 5′-GCCGATGATCTCTCTCAAGTGA-3′; mouse IL-13, sense 5′-CCTGGCTCTTGCTTGCCTT-3′ and antisense 5′-GGTCTTGTGTGATGTTGCTCA-3′; mouse TNF-*α*, sense 5′-CTGTAGCCCACGTCGTAGC-3′ and antisense 5′-TTGAGATCCATGCCGTTG-3′; and mouse *β*-actin sense 5′-ATGCCATCCTGCGTCTGGACCTGGC-3′ and antisense 5′-AGCATTTGCGGTGCACGATGGAGGG-3′. The denaturation, annealing, and extension conditions and number of cycles run were 94°C for 60 s, 49°C for 45 s, and 72°C for 45 s for 35 cycles, respectively.

### 2.10. Statistical Analysis

The experimental results are expressed as the mean ± standard deviation (SD). A one-way analysis of variance (ANOVA) was used for multiple comparisons followed by a Tukey-Kramer post hoc analysis. *P* values < 0.05, <0.001, and <0.0001 were considered statistically significant.

## 3. Results

### 3.1. Effect of CYM on IgE/DNP-BSA-Induced Passive Cutaneous Anaphylaxis

**Figure 1:**
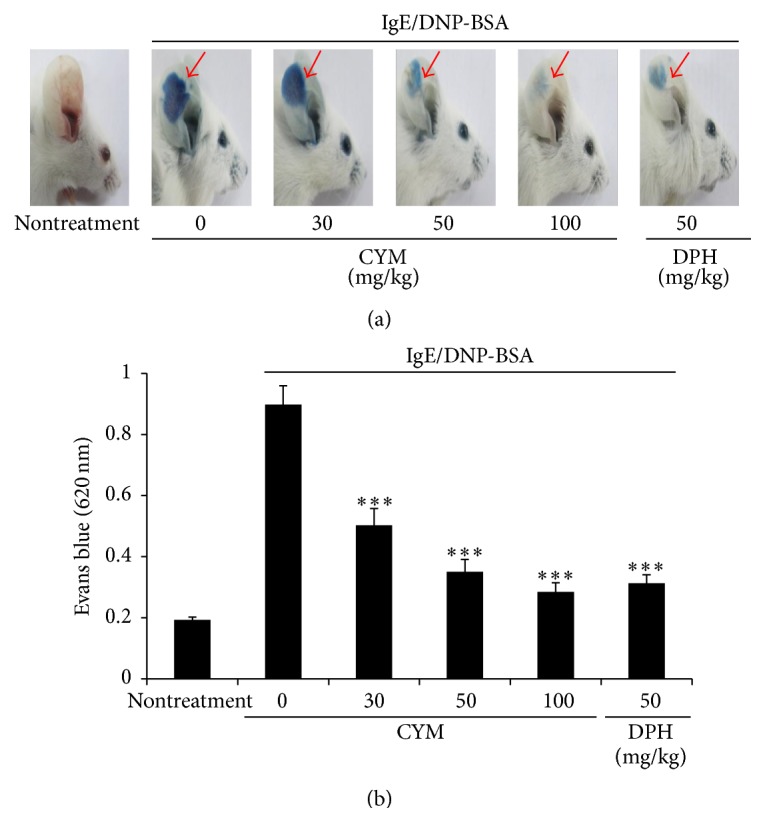
Effect of* Cymbidium* ethanol extract (CYM) on immunoglobulin E/dinitrophenyl-conjugated bovine serum albumin- (IgE/DNP-BSA-) induced passive cutaneous anaphylaxis. Anti-DNP IgE antibody was injected intradermally into each right ear of 7-week-old ICR mice. CYM or diphenhydramine (DPH) was administered orally to mice. After 1 h, we injected 200 *μ*g of DNP-BSA. (a) Representative images of ears. (b) Excised ears were reacted overnight with 500 *μ*L of formamide and amount of dye taken up was determined by measuring absorbance at 620 nm using a microplate reader. Data are mean ± standard deviation (SD, *n* = 5); ^*∗∗∗*^ *P* < 0.0001 versus IgE/DNP-BSA treatment group.

Passive cutaneous anaphylaxis was investigated to determine whether CYM affected local allergic responses* in vivo*. We locally sensitized the ears of mice by injecting them with IgE, followed by intravenous injection of DNP-BSA and Evans Blue solution. The CYM treatment group showed a suppressed local allergic response, which was similar to that observed after treatment with DPH, an antihistamine (Figures 1(a) and 1(b)).

### 3.2. Effect of CYM on DNCB-Induced Dermatitis in an AD-Like Mouse Model

**Figure 2:**
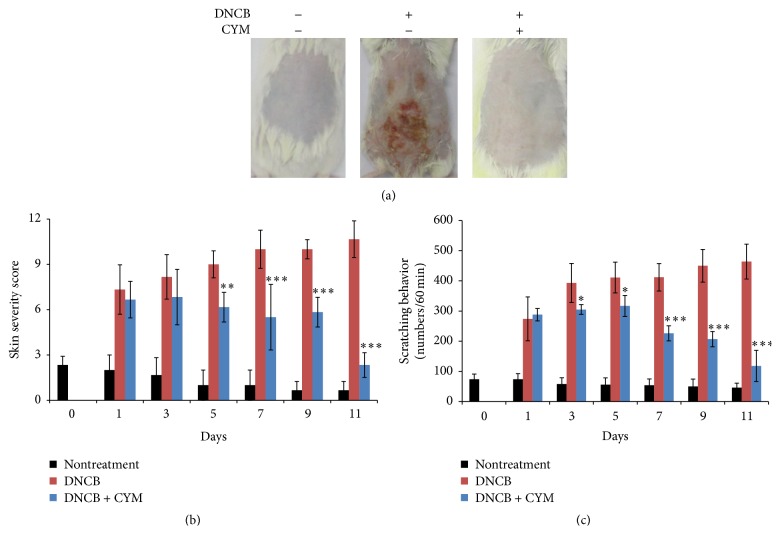
Effect of* Cymbidium* ethanol extract (CYM) on 2,4-dinitrochlorobenzene- (DNCB-) induced atopic dermatitis- (AD-) like model. Male ICR mice were divided into three groups (non-, DNCB, and DNCB and CYM treatment, *n* = 6). AD was induced by shaving dorsal skin of mice and applying a 1% DNCB solution for 4 days. After sensitization, mice were treated with 0.5% DNCB solution with or without CYM (10 mg/mL) every 2 days for a total of 6 times. Subsequently, AD-like mice were euthanized. (a) Image of mouse dorsal skin after euthanasia. (b) AD-like mouse model dorsal skin lesion conditions were evaluated to determine severity of dermatitis. Five symptomatic conditions were evaluated, erythema, pruritus/dry skin, erosion, lichenification, and edema/excoriation, and scored based on severity as follows: none = 0, mild = 1, moderate = 2, and severe = 3. Sum of individual scores was defined as dermatitis score. (c) Behavior of AD-like mice was video recorded and the number of scratching behaviors was measured. Data are mean ± standard deviation (SD, *n* = 6). Days on *x*-axis are after sensitization. ^*∗*^ *P* < 0.05, ^*∗∗*^ *P* < 0.001, and ^*∗∗∗*^ *P* < 0.0001 versus DNCB treatment.

Continuous DNCB treatment induced dermatitis in mice while CYM treatment reversed this AD-like effect (Figure 2(a)). To evaluate the condition of the mouse skin lesions, we measured skin severity scores for 11 days following sensitization. The continuous DNCB treatment not only aggravated the condition associated with the lesions on the dorsal skin, such as erythema, pruritus/dry skin, erosion, lichenification, and edema/excoriation but also increased the severity scores. However, CYM treatment improved the skin conditions 5 days following sensitization (Figure 2(b)). Scratching behavior was measured using 60 min video recordings of the mouse behaviors for 11 days following the sensitized AD-like skin severity test. During the behavioral observations, the blinded researcher counted the number of scratching behaviors, and, as shown in Figure 2(c), the group with continuous DNCB treatment exhibited an increase in the number of scratches to the dorsal skin. However, treatment with CYM decreased scratching behavior 7 days following sensitization.

### 3.3. Effect of CYM on DNCB-Induced Histopathological Changes and IgE Level in an AD-Like Mouse Model

**Figure 3:**
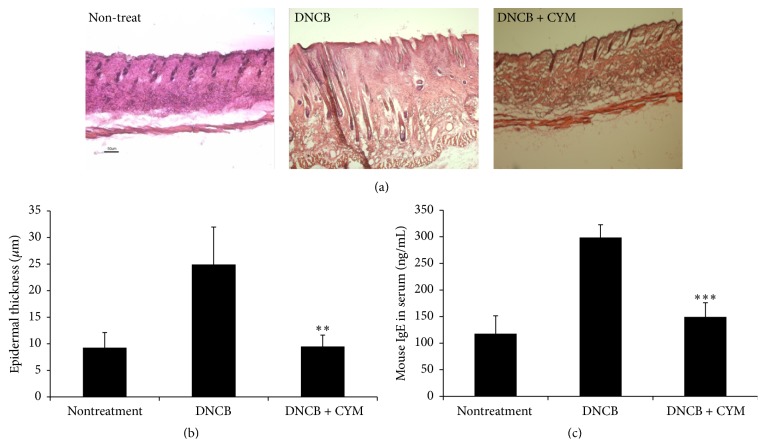
Effect of* Cymbidium* ethanol extract (CYM) on 2,4-dinitrochlorobenzene- (DNCB-) induced histopathological changes and immunoglobulin E (IgE) level in atopic dermatitis- (AD-) like mouse model. After inducing AD, 10 mg/mL CYM solution (in 3 : 1 mixture of acetone and olive oil) was applied to the dorsal skin of mice for a total of 6 times over a 2-week period. (a) After euthanasia, dorsal skin lesions were enucleated from mice in all three groups (non-, DNCB, DNCB, and CYM treatment), fixed, cryoprotected, sectioned, and stained with hematoxylin and eosin (H&E). (b) Densitometric analysis of epidermal thickness of each group was performed. To measure thickness of tissues, densitometric analysis was performed using a Nikon light microscope and an associated program. Data are mean ± standard deviation (SD, *n* = 4); ^*∗∗*^ *P* < 0.001 versus DNCB treatment. (c) Serum IgE was analyzed using a mouse IgE kit and was determined relative to standard absorbance values measured at 450 nm using a microplate reader. Data are mean ± SD (*n* = 6); ^*∗∗∗*^ *P* < 0.0001 versus DNCB treatment.

In AD, the thickness of skin lesions increases owing to the inflammatory condition, and serum IgE levels are upregulated. To measure skin thickness, enucleated skin lesion tissue was H&E-stained. Continuous DNCB treatment resulted in thickening of the epidermis and dermis of the dorsal skin compared with that of the nontreatment group. CYM treatment not only reduced the thickening of the dorsal skin but also resulted in a dorsal skin thickness that was similar to that of the nontreatment group (Figures 3(a) and 3(b)). The IgE serum levels were measured using a mouse IgE kit and the group that was continuously treated with DNCB exhibited an increased level. However, serum IgE levels of the CYM treatment group were effectively restored to levels that were similar to those of the nontreatment group (Figure 3(c)).

### 3.4. Effect of CYM on DNCB-Induced mRNA Expression of IL-4, IL-13, and TNF-*α* in AD-Like Mouse Model

**Figure 4:**
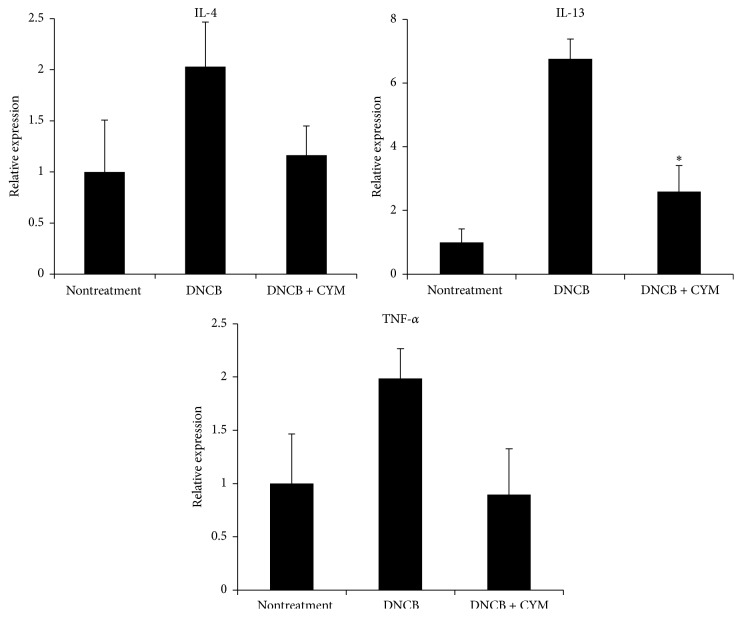
Effect of* Cymbidium* ethanol extract (CYM) on 2,4-dinitrochlorobenzene- (DNCB-) induced expression of interleukin- (IL-) 4, IL-13, and tumor necrosis factor- (TNF-) *α* mRNA in atopic dermatitis- (AD-) like mouse model. After inducing AD, 10 mg/mL CYM solution (in 3 : 1 mixture of acetone and olive oil) was applied to the dorsal skin of mice for a total of 6 times over a 2-week period. After euthanasia, dorsal skin lesions were enucleated from mice of all three groups (non-, DNCB, DNCB, and CYM treatment). Total RNA was isolated from extracted tissue of dorsal lesions using Tri-reagent. Total RNA was used as a template for cDNA synthesis, which was performed using a cDNA Synthesis kit. Real-time polymerase chain reaction (qPCR) analysis was carried out using SYBR Green I and a Lightcycler 96 instrument. The qPCR analysis was performed to detect IL-4, IL-13, and TNF-*α* mRNA expression. Data are mean ± standard deviation (SD, *n* = 6). ^*∗*^ *P* < 0.05 versus DNCB treatment.

Proinflammatory cytokines such as* IL-4, IL-13, and TNF-α* are known to induce inflammatory reactions. Therefore, the gene expression of these cytokines was analyzed using qPCR. As shown in Figure 4, the expression of mRNA increased in the DNCB treatment group while CYM efficiently suppressed this effect by 80%, 400%, and 90% for* IL-4, IL-13,* and* TNF-α,* respectively, compared with DNCB treatment.

### 3.5. Effect of CYM on Viability and IgE/DNP-BSA-Induced Degranulation of RBL-2H3 Cells

**Figure 5:**
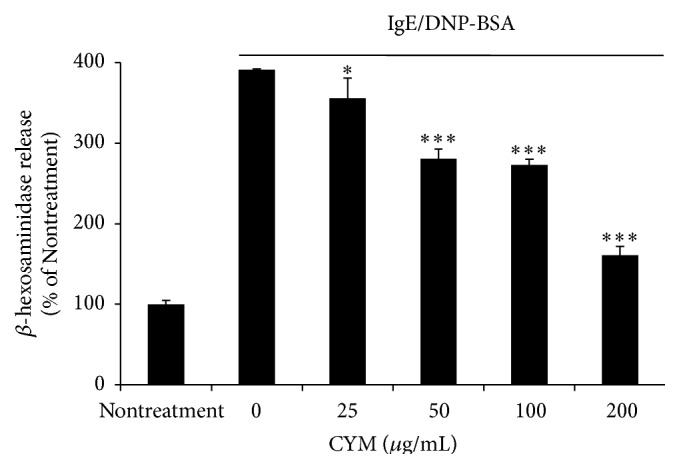
Effect of* Cymbidium* ethanol extract (CYM) on viability and immunoglobulin E/dinitrophenyl-conjugated bovine serum albumin- (IgE/DNP-BSA-) induced degranulation of RBL-2H3 cells. RBL-2H3 cells were seeded and incubated in 24-well plates overnight in Eagle's Minimal Essential Medium (MEM). To sensitize RBL-2H3 cells, anti-DNP IgE (400 ng/mL) was added to culture medium. After 12 h, RBL-2H3 cells were washed with Siraganian buffer and treated with CYM (0, 25, 50, 100, or 200 *μ*g/mL) in Siraganian buffer for 30 min. DNP-BSA (50 ng/mL) was added for 15 min. Cell supernatants were transferred to a 96-well plate, mixed with 1 mM p-NAG in 0.1 M citrate buffer for 1 h, and absorbance was measured at 405 nm using a microplate reader. Data are mean ± standard deviation (SD, *n* = 3). ^*∗*^ *P* < 0.05 and ^*∗∗∗*^ *P* < 0.0001 versus sensitized RBL-2H3 cells.

IgE/DNP-BSA-induced RBL-2H3 cell degranulation was measured using a *β*-hexosaminidase release assay. *β*-Hexosaminidase is a lysosomal enzyme and was used as a marker of RBL-2H3 cell degranulation in this study. IgE/DNP treatment induced a 4-fold release of *β*-hexosaminidase compared with the nontreatment group. The inhibitory effect of CYM on degranulation of RBL-2H3 cell was concentration-dependent. At the maximum dose (200 *μ*g/mL) of CYM, the inhibitory effect increased by 230% compared with IgE/DNP-BSA treatment (Figure 5). In addition, CYM was not toxic to RBL-2H3 cells at concentrations of up to 200 *μ*g/mL (data not shown).

### 3.6. Effect of CYM on IgE/DNP-BSA-Induced Phosphorylation of Syk, PLC-*γ*, Akt, Erk 1/2, JNK, and p38

**Figure 6:**
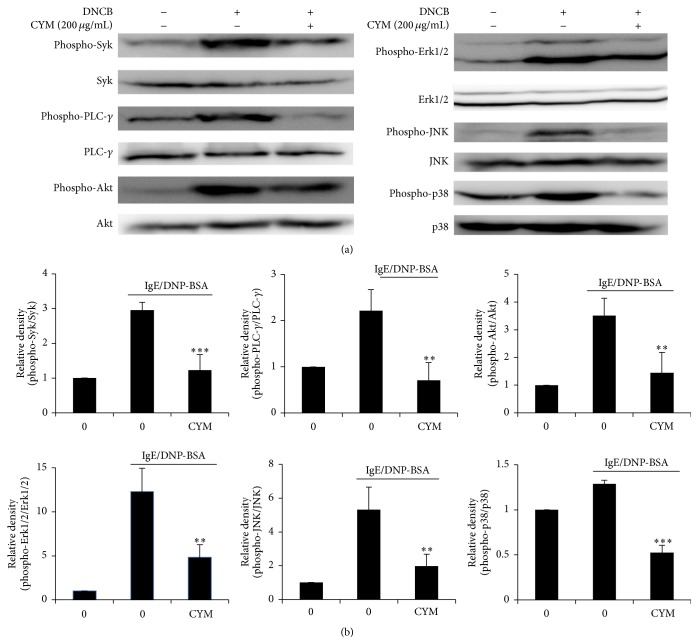
Effect of* Cymbidium* ethanol extract (CYM) on immunoglobulin E/dinitrophenyl-conjugated bovine serum albumin- (IgE/DNP-BSA-) induced phosphorylation of spleen tyrosine kinase (Syk), phospholipase C- (PLC-) *γ*, Akt, extracellular signal-regulated kinase (Erk) 1/2, c-Jun N-terminal kinase (JNK), and p38. RBL-2H3 cells were seeded and incubated in 24-well plates overnight in Minimum Essential Medium (MEM). Anti-DNP IgE (400 ng/mL) was added for 12 h, followed by addition of CYM (0 or 200 *μ*g/mL). Cells were then treated with DNP-BSA. Cells were lysed and protein was extracted by the PRO-PREP protein extraction kit. The protein lysates were analyzed by SDS-PAGE. (a) Western blot analysis for phosphorylation of Syk, PLC-*γ*, Akt, Erk1/2, JNK, and p38. (b) Densitometric analysis of expression of phosphorylated proteins. Data are mean ± standard deviation (SD, *n* = 3); ^*∗∗*^ *P* < 0.001 and ^*∗∗∗*^ *P* < 0.0001 versus sensitized RBL-2H3 cell.

The effect of CYM on specific signal transduction pathways was determined using western blot analysis. Signal transduction is usually initiated by extracellular signaling via receptors on the surface or inside the cell. IgE/DNP-BSA activates RBL-2H3 cells, thereby initiating signaling cascades. We investigated the phosphorylated signaling molecules related to RBL-2H3 cell degranulation. The phosphorylation of Syk is mediated by early Fc*ε*R1 signaling. The phosphorylation of Syk was decreased by CYM treatment by about 2-fold compared to that observed after IgE/DNP-BSA treatment. The PLC-*γ*-induced cleavage of phospholipids is related to calcium influx. The phosphorylation of PLC-*γ* was strongly decreased by about 3-fold by CYM treatment group compared to that observed after IgE/DNP-BSA treatment. CYM treatment also suppressed the phosphorylation of Akt by about 2.5-fold compared with IgE/DNP-BSA treatment. The Erk 1/2, JNK, and p38 proteins, which are subfamilies of MAPK, and their phosphorylation were suppressed by about 3-, 2.7-, and 2-fold, respectively, by CYM treatment (Figures 6(a) and 6(b)).

## 4. Discussion

The allergic response is related to mast cells, which are derived from myeloid stem cells. Fc*ε*R1 is located on the surface of mast cells and is a binding site for IgE [13–15]. In the presence of an allergen, the cross-linking of IgE and the allergen initiates mast cell activation [16]. Mast cells have a number of granules, and their activation induces not only degranulation but also the secretion of various factors such as histamine, leukotriene, prostaglandins, and other mediators from granules to various tissues and cells [20, 21]. They also express proinflammatory cytokines such as IL-4, IL-13, and TNF-*α* that induce inflammatory reactions [22, 23]. Here, IgE/DNP-BSA initiated the allergic response* in vivo* while CYM had an inhibitory effect on IgE/DNP-BSA-induced anaphylactic shock as shown by the PCA test (Figure 1).

Mast cells play critical roles in the allergic response and release a number of biologically active substances. To identify the therapeutic effects of CYM, we investigated the effect of DNCB induction in an AD-like mouse model [28, 29]. The continuous application of DNCB significantly increased the symptoms of dermatitis. However, CYM had therapeutic effects on DNCB-induced skin lesions in AD-like mice. In the severity test, the continuous application of DNCB aggravated the dorsal skin conditions such as erythema, pruritus/dry skin, erosion, lichenification, and edema/excoriation. As the inflammatory reaction worsened, the dorsal skin thickened. The application of DNCB not only increased the scratching behavior, which is a typical behavior in allergic dermatitis, but also abnormally upregulated the IgE levels in the serum of AD-like mice [30, 31]. CYM efficiently suppressed the increased skin severity, scratching behavior, epidermal thickness, and IgE levels (Figures 2 and 3). Additionally, CYM suppressed the gene expression of the proinflammatory cytokines IL-4, IL-13, and TNF-*α* (Figure 4) [23].

**Figure 7:**
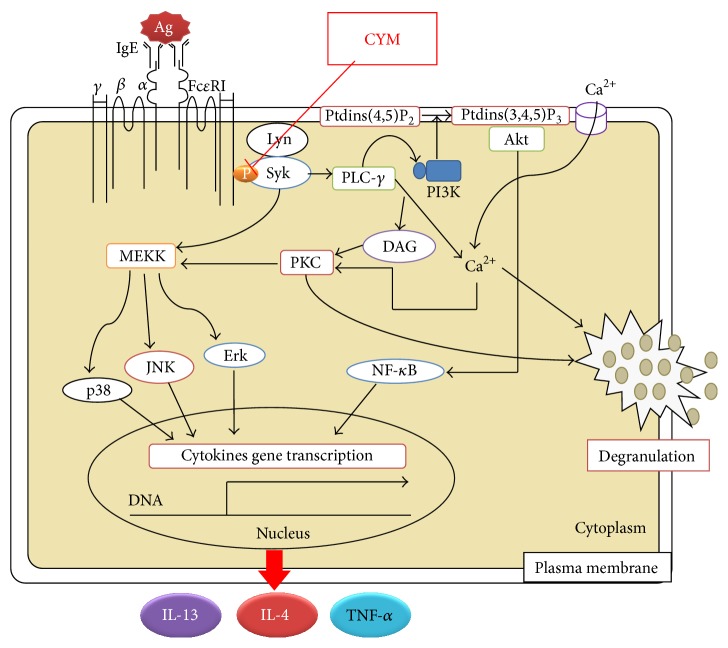
*Cymbidium* ethanol extract (CYM) suppresses immunoglobulin E/dinitrophenyl-conjugated bovine serum albumin- (IgE/DNP-BSA-) stimulated RBL-2H3 cell activation via spleen tyrosine kinase- (Syk-) mediated signal transduction in RBL-2H3 cells.

The initiation of an allergic response is followed by associated symptoms such as AD, anaphylactic shock, asthma, and conjunctivitis [4, 5]. CYM had an inhibitory effect on degranulation as shown in the *β*-hexosaminidase release assay (Figure 5). The degranulation of RBL-2H3 cells was controlled by cascades of multiple tyrosine kinases, and Syk is phosphorylated early in Fc*ε*R1-mediated signaling. Syk phosphorylation activates signal transduction pathways such as the phosphoinositide 3-kinase (PI3K)/AKT and the MAPK pathways [1, 17]. Phosphorylation of PLC-*γ*, PIP2, DAG, and IP3 occurs following calcium ion influx into the cytosol through calcium channels [18]. An increased level of calcium ions in the cytoplasm also induces the degranulation of RBL-2H3 cells. The Akt and MAPK pathways regulate transcriptional activity. Consequently, phosphorylation of Akt, Erk 1/2, JNK, and p38 induces transcription of proinflammatory cytokine genes such as* IL-4, IL-13,* and* TNF-α*. CYM was effective in inhibiting the degranulation of RBL-2H3 cells by suppressing the phosphorylation of various proteins such as Syk and PLC-*γ* and that of proinflammatory cytokines through the inhibition of phosphorylation of Akt, Erk 1/2, JNK, and p38 (Figure 6). Therefore, CYM reduced AD by suppressing Syk-mediated degranulation of RBL-2H3 cells and gene expression of cytokines (Figure 7).

## 5. Conclusion

In conclusion, CYM had an antiallergic effect on dermatitis by inhibiting Syk-mediated signal transduction, suppressing proinflammatory cytokine gene expression and reducing serum IgE levels. We suggest that CYM may have therapeutic potential as an antiallergic agent for treating disorders such as AD.
